# A meta‐analysis of the effectiveness of Yangxue Qingnao granules for the treatment of chronic cerebral circulation insufficiency

**DOI:** 10.1002/brb3.1606

**Published:** 2020-03-31

**Authors:** Yujuan Jia, Jie Wang, Yuli Hou

**Affiliations:** ^1^ Department of Neurology First Hospital of Shanxi Medical University Taiyuan China

**Keywords:** adverse effects, chronic cerebral circulation insufficiency, effectiveness, meta‐analysis, Yangxue Qingnao granules

## Abstract

**Objective:**

Yangxue Qingnao granules (YXQNG), which translates to granules that tonify the blood and clear liver heat, are widely available in China for the treatment of Chronic Cerebral Circulation Insufficiency (CCCI). This systematic review aimed to evaluate the effectiveness and safety of YXQNG in treating CCCI.

**Methods:**

PubMed, the Cochrane Central Register of Controlled Trials, Embase, the Chinese National Knowledge Infrastructure, and the Wanfang Database were searched from their inception to February 2019. Randomized controlled trials of YXQNG used alone or in combination with other drugs against a placebo, with no intervention or used with other drugs in CCCI patients were identified. The reviewers identified studies, extracted data, and assessed the quality of the evidence, independently and in duplicate. The Cochrane risk of bias assessment tool was used for quality assessment.

**Results:**

A total of 31 RCTs and 2,877 patients were selected. The meta‐analysis indicated that the ratio between the combined RR of the total effective rate and the 95% confidential interval (95% CI) was 1.21 (1.17, 1.26). The combined MD of transcranial Doppler (TCD) detecting carotid artery, vertebral artery, and basilar artery blood flows (95% CI), respectively, were 8.84 (5.83, 11.85), 4.72 (3.71, 5.73), and 3.89 (3.03, 4.76). The combined MD of plasma viscosity and fibrinogen, respectively, were −0.35 (−0.40, −0.30) and −0.81 (−1.12, −0.50). Serious adverse effects were not reported in all the included trials.

**Conclusion:**

This systematic review revealed that YXQNG could increase cerebral blood flow in patients with CCCI and improve their symptoms, with no serious adverse effects. Since the literature reviewed was affected by factors such as lower quality of the included studies, the systematic evaluation's conclusion is not very reliable. Thus, more rigorously designed trials are needed.

## Graphical abstract

Yangxue Qingnao granules (YXQNG), which translates to granules to tonify blood and clear liver heat, are widely available in China for treating Chronic Cerebral Circulation Insufficiency (CCCI). This systematic review aims to evaluate the effectiveness and safety of YXQNG in treating CCCI.

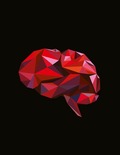

## 1 INTRODUCTION

Chronic cerebral circulation insufficiency (CCCI), initially proposed by Japanese scholars in the 1990s, refers to a state of persistent reduction in cerebral blood flow (CBF), resulting in brain dysfunctions (Calabrese et al., 2016). CCCI may not be an independent disease; rather, it is a syndrome caused by a variety of etiologies. Thus, CCCI is considered to be related to either the occurrence or recurrence of ischemic stroke, vascular cognitive impairment, and the development of vascular dementia, leading to serious disability and mortality (Chang, 2013). Previous clinical studies have suggested that the symptoms of CCCI are actually reversible once cerebral circulation is improved, such as dizziness, headache, and so on (Dong, 2015; Feng, 2018). On the contrary, long‐term CBF reduction, namely CCCI, may evoke stroke, TIA, vascular cognitive impairment, or even dementia (Calabrese et al., 2016; Chang, 2013; Dong, 2015; Feng, 2018). Thus, identifying these syndromes followed by effective treatment is enormously valuable.

However, so far, there is no effective clinical treatment available for CCCI. Yangxue Qingnao granules (YXQNG), which have the effect of tonifying the blood and clearing liver heat, were widely used to treat symptoms caused by blood deficiency and excessive liver yang, such as headaches, vertigo, insomnia, dizziness, as well as vascular dementia (Chang, 2013). According to these reports, YXQNG was suggested for the treatment of CCCI (Calabrese et al., 2016). Although several studies revealed that YXQNG could reduce the symptoms of CCCI, the number of patients enrolled in these previous studies was small and the conclusion was not very reliable (Gu & Cai, 2005). The objective of this current meta‐analysis was to better evaluate the efficacy of YXQNG for increasing cerebral blood flow and improving CCCI‐related symptoms.

## 2 METHODS

### 2.1 Study selection

#### 2.1.1 The types of studies

All the RCTs reporting the application of YXQNG for the treatment of CCCI were involved with no limitations on language and publication.

#### 2.1.2 The types of participants

All the participants in the included studies had to meet the diagnostic criteria of CCCI, which was proposed by Japanese scholars in 2000 (Gu et al., 2005). The diagnostic criteria included: (i) The presence of one or more of these symptoms: dizziness, especially positional dizziness, vertigo, heavy‐headed feeling and/or headache; (ii) The presence of cerebrovascular stenosis, especially fundus oculi arteriosclerosis, revealed by transcranial Doppler; and (iii) The absence of organic cerebrovascular diseases judged with computed tomography or magnetic resonance imaging (Chang, 2013).

Exclusion criteria included: (i) Patients with a cerebral infarction; (ii) Patients diagnosed with depression or other psychiatric disorders; and (iii) Patients with clinically relevant pulmonary, hepatic, gastrointestinal, or life‐threatening diseases. No limitations on age, gender, or ethnicity of the patients were set.

#### 2.1.3 The types of interventions

The patients were randomized into either a YXQNG group or a control group. RCTs comparing YXQNG versus placebo or no intervention were included. Studies comparing YXQNG against other drugs aimed to treat CCCI were also included. The other drugs aimed to treat CCCI had to be given identically to both the YXQNG group and the control group. The treatment duration was required to be at least 2 weeks.

#### 2.1.4 The types of outcome measures

The primary endpoint was the overall effectiveness, defined as the percentage of patients with improved symptoms. The efficacy of YXQNG on the change in the patients’ symptoms was classified into four grades: (i) The symptoms and signs disappeared basically; (ii) The symptoms and signs significantly improved; (iii) The symptoms and signs improved; and (iv) The symptoms and signs did not improve. The secondary outcomes were the changes in blood viscosity and the cerebral blood flow rate revealed by transcranial Doppler.

### 2.2 Search strategy

We searched PubMed, the Cochrane Central Register of Controlled Trials, Embase, the Chinese National Knowledge Infrastructure, and the Wanfang Database from their inception to February 2019 for the relevant randomized controlled trials (RCTs). We also searched for unpublished clinical trials on two trial registries (://www.clinicaltrials.gov/ and ://www.chictr.org/). There were no limitations on language or publication status. The search terms included: (“Chronic Cerebral Circulation Insufficiency” OR “chronic cerebrovascular insufficiency” OR “CCCI” OR “man xing nao gong xue bu zu”) AND (“Yangxue Qingnao Granules” OR “Yangxue Qingnao Keli”) AND (“randomized controlled trial” OR “clinical trial”).

### 2.3 Data extraction

The eligible studies were independently screened by two reviewers and in duplicate based on the titles, the abstracts, and the full text. Then, they resolved the discrepancies by discussion or by a third reviewer. The basic characteristics of the included studies were extracted, including the first author's name, year of publication, the number of participants, age, interventions for YXQNG and the control groups, the duration of treatment, and outcome measures.

### 2.4 The assessment of the risk of bias

Two reviewers independently, and in duplicate, evaluated the risk of bias of the eligible studies according to the assessment tool of the Cochrane Handbook (He, Ming, & Hang, 2013). The criteria included: (1) The sequence generation; (2) The allocation concealment; (3) The blinding of participants and personnel; (4) The blinding of outcome assessments; (5) Incomplete outcome data; (6) Selective reporting; and (7) Other sources of bias.

### 2.5 Data analysis

A meta‐analysis was conducted using Review Manager 5.3. The risk ratio (RR) with a 95% CI was used for the improvement of symptoms, while the weighted mean difference (WMD) with a 95% CI was adopted for the changes of blood viscosity and cerebral blood flow rate revealed by transcranial Doppler (TCD). Heterogeneity was assessed by the visual inspection of forest plots, *p*‐values, and *I* ^2^ statistics. *p*> .10 and *I* ^2^ < 50% suggested a lack of significant heterogeneity; in such cases, a fixed‐effects model was used for the meta‐analysis. For cases with *p* < .10 and *I* ^2^> 50%, we explored sources of heterogeneity. When no clinical heterogeneity was indicated, a random‐effects model was used to perform the meta‐analysis. A value of *p *< .05 was considered to be statistically significant.

A funnel plot was used to assess publication bias. An Egger test and Begg test were also used to analyze the potential publication bias. A value of *p*> .05 meant that the risk of publication bias was small, while *p* < .05 meant that publication bias might exist.

## 3 RESULTS

### 3.1 Study identification

**Figure 1:**
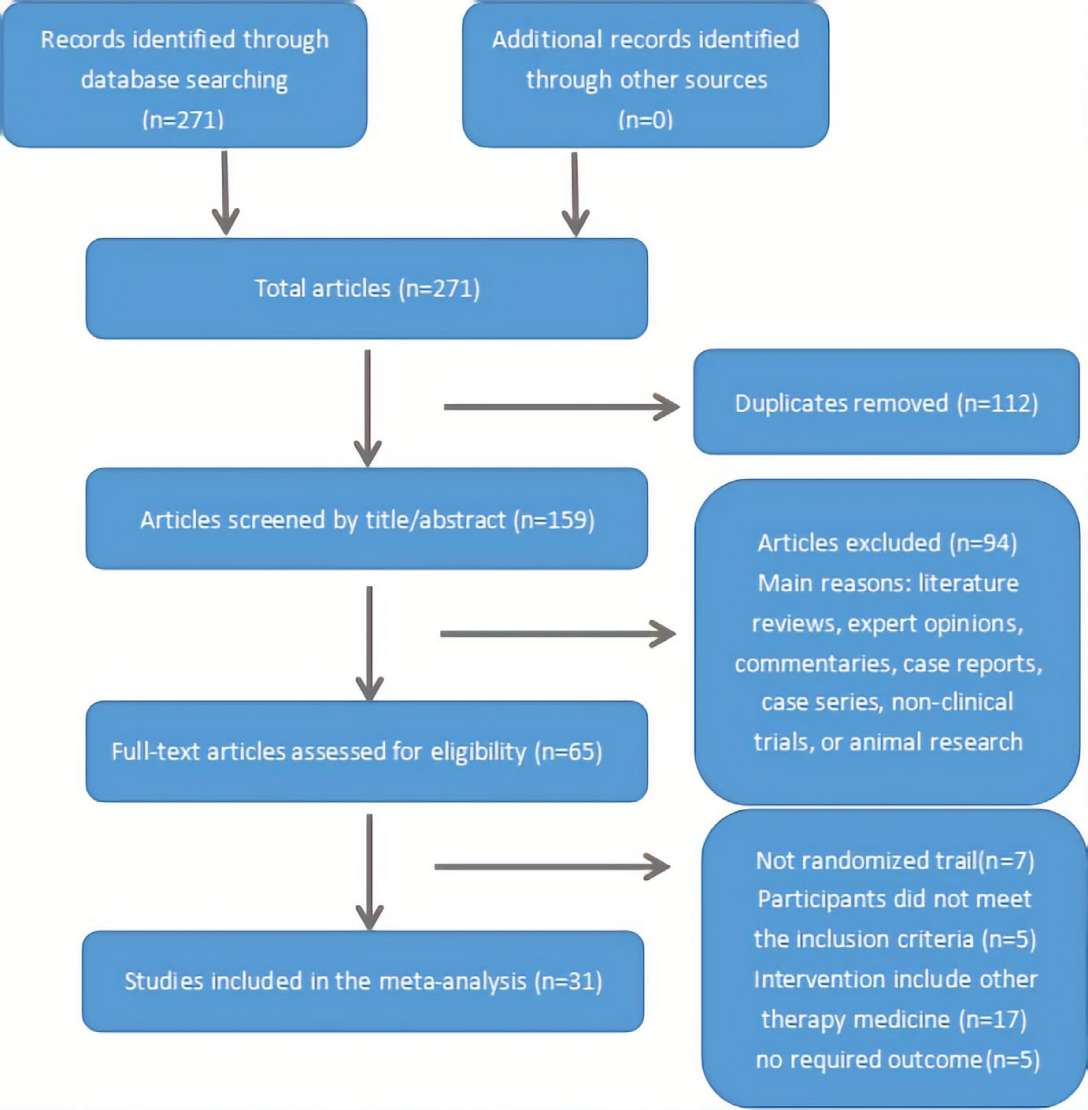
Flow diagram of the study selection and identification

Figure 1 shows the detailed process of the study selection. A total of 271 potentially relevant studies were initially screened. A total of 159 titles and abstracts were identified through the literature search, of which 94 studies were excluded because they were literature reviews, commentaries, expert opinions, nonclinical trials, case series, case reports, or animal research. The remaining 65 full‐text studies were potentially eligible and 34 were excluded for the reasons as follows: not a randomized trial (*n* = 7), the patients did not meet the inclusion criteria (*n* = 5), the intervention included other medical therapies (*n* = 17), and there were no required outcomes (*n* = 5).

### 3.2 Study characteristics

The basic characteristics of the 31 included randomized trials are summarized in Table 1. A total of 2,877 CCCI patients were enrolled, with 1,458 in the YXQNG group and 1,419 in the control group. All the studies were conducted in China and all the patients involved were Chinese. The sample size of all the eligible studies was small and ranged from 48 to 200 patients.

**Table 1:** The basic characteristics of the included studies

Studies	Sample size (E/C)	Age (E/C) (years)	Intervention	Control	Treatment duration (E/C)	Outcomes
Chang, (2013)	26/26	62.5 ± 4.6/61.3 ± 4.2	YXQNG + Conventional treatment	Conventional treatment	12 weeks	①
Dong (2015)	36/36	61.6 ± 8.2/60.8 ± 9.8	YXQNG + Conventional treatment	Conventional treatment	30 days	①
Feng (2018)	54/54	61.7 ± 6.5/61.3 ± 6.9	YXQNG + Conventional treatment	Conventional treatment	3 months	①
Gu (2005)	42/41	67.2/69.2	YXQNG	nimodipine	8weeks	②
He (2013)	34/31	63.3 ± 5.6/63.7 ± 7.4	YXQNG + Conventional treatment	Conventional treatment	30 days	①
Jiao (2018)	100/100	61.7 ± 6.5/61.3 ± 6.9	YXQNG + Conventional treatment	Conventional treatment	3 months	①
Lei (2011)	40/38	66.8 ± 5.1/67.8 ± 5.3	YXQNG + Conventional treatment	Conventional treatment	14 days	①
Li (2011)	24/24	60–82	YXQNG + Conventional treatment	Conventional treatment	30 days	①
Li (2013)	26/26	86.6/84.6	YXQNG + Conventional treatment	Conventional treatment	90 days	①
Li (2015)	43/43	65.9 ± 7.1/64.8 ± 7.6	YXQNG + Conventional treatment	Conventional treatment	30 days	①
Li (2018)	100/100	74.23 ± 3.67/73.54 ± 3.56	YXQNG + Conventional treatment	Conventional treatment	3 months	①
Liu (2014)	25/25	62.8 ± 6.5/62.5 ± 6.7	YXQNG + Conventional treatment	Conventional treatment	1 month	①③
Liu et al. (2017)	50/50	55.11 ± 3.65/55.25 ± 3.71	YXQNG + Conventional treatment	Conventional treatment	1 month	①
Peng et al. (2014)	40/40	69.8/70.1	YXQNG + Conventional treatment	Conventional treatment	12 weeks	①
Shao (2016)	30/30	63.0 ± 2.2/62.4 ± 1.8	YXQNG + Conventional treatment	Conventional treatment	30 days	①③
Shen (2010)	52/50	58 ± 6. 8/57 ± 7.8	YXQNG + Conventional treatment	Conventional treatment	1 month	①②
Song (2012)	100/98	65.9 ± 6.3/67.5 ± 5.6	YXQNG + Conventional treatment	Conventional treatment	30 days	①②③
Sun (2015)	51/51	54. ± 11.7/60.7 ± 9.8	YXQNG + Conventional treatment	Conventional treatment	1 month	①
Wang (2010)	44/43	62 ± 7.5/61. 5 ± 8.5	YXQNG + Conventional treatment	Conventional treatment	1 month	①②
Wang (2014)	68/58	63.2 ± 6.6/63.8 ± 6.4	YXQNG + Conventional treatment	Conventional treatment	4 weeks	①
Wang (2017)	37/37	54.22 ± 3.23/54.25 ± 3.21	YXQNG + Conventional treatment	Conventional treatment	14 days	①②
Xue (2015)	40/36	63.3 ± 3.6/64.2 ± 4.2	YXQNG + Conventional treatment	Conventional treatment	15 days	①
Yang (2010)	35/35	64.1 ± 4.5/63.2 ± 5.2	YXQNG + Conventional treatment	Conventional treatment	15 days	①
Yang (2016)	30/30	69.2 ± 6.8/68.4 ± 7.2	YXQNG + Conventional treatment	Conventional treatment	2 months	①③
Yang (2017)	58/58	65.71 ± 8.26/65.98 ± 8.17	YXQNG + Conventional treatment	Conventional treatment	14 days	①
Zhang (2008)	56/48	66.4 ± 6.2/63.2 ± 5.3	YXQNG + Conventional treatment	Conventional treatment	30 days	①
Zhao (2014)	55/55	58.12 ± 8.78	YXQNG + Conventional treatment	Conventional treatment	8 weeks	①③
Zhou (2017)	30/30	61.6 ± 8.2/60.8 ± 9.8	YXQNG + Conventional treatment	Conventional treatment	30 days	①②
Zhou (2008)	46/40	46 ± 2.0/48 ± 1.8	YXQNG + Conventional treatment	Conventional treatment	4 weeks	①
Zhu (2006)	38/38	80.2/79.6	YXQNG + Conventional treatment	Conventional treatment	8 weeks	①
Zhu (2011)	48/48	50–75	YXQNG + Conventional treatment	Conventional treatment	2 weeks	①③

①:the change from baseline in severity of symptoms; ②:the change of cerebral blood flow rate revealed by transcranial Doppler; ③:the change of blood viscosity.

All the included studies were two‐arm designed RCTs. For the interventions, the patients in the treatment group received YXQNG, while the patients in the control group received a placebo, no intervention, or other drugs (as shown in Table 1). The primary outcome was reported in all of the studies. The changes in blood viscosity and cerebral blood flow rate were, respectively, reported in 5 and 7 studies. The treatment duration ranged from 2 weeks to 90 days.

### 3.3 Risk of bias

**Figure 2:**
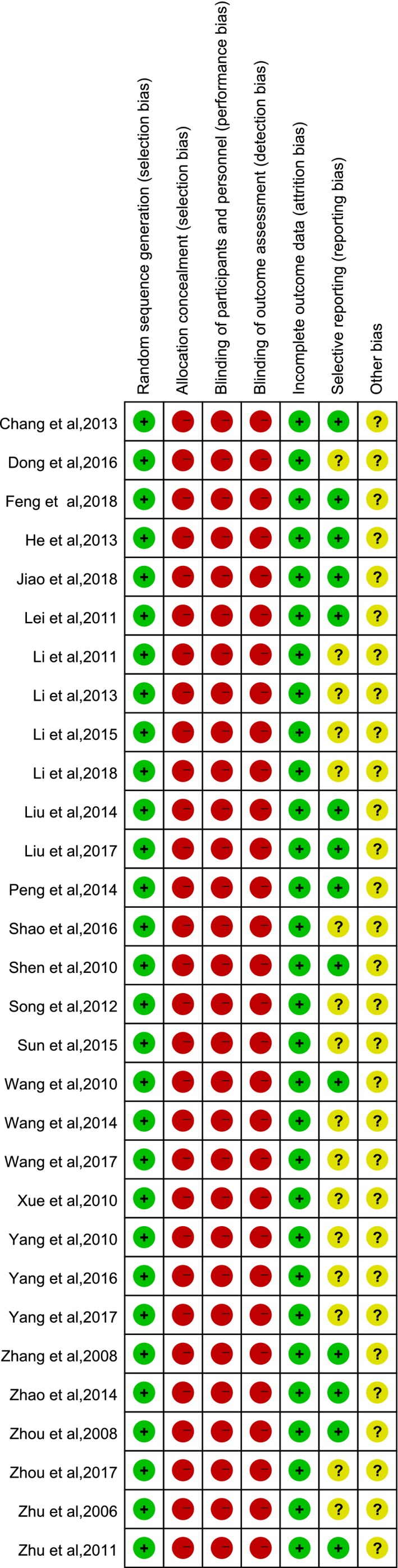
Risk of bias summary + low risk; ‐ high risk; unclear risk

Figure 2 shows the details of the risk of bias in the included studies. Randomization was declared in all the studies. None of the studies described allocation concealment, the blinding of participants and personnel, and the blinding of outcomes assessment; therefore, the risk of bias was assessed as high. None of the studies had incomplete outcome data, leading to a low risk of detection bias. Among all the included studies, 14 trials were evaluated as low risk of reporting bias because they reported both the curative effects and adverse effects objectively, while the other 16 studies that only reported the effectiveness were assessed as unclear. The other bias was evaluated as unclear because no other information could be obtained.

### 3.4 Outcome measures

#### 3.4.1 Primary outcome: the change from baseline in the severity of symptoms

**Figure 3:**
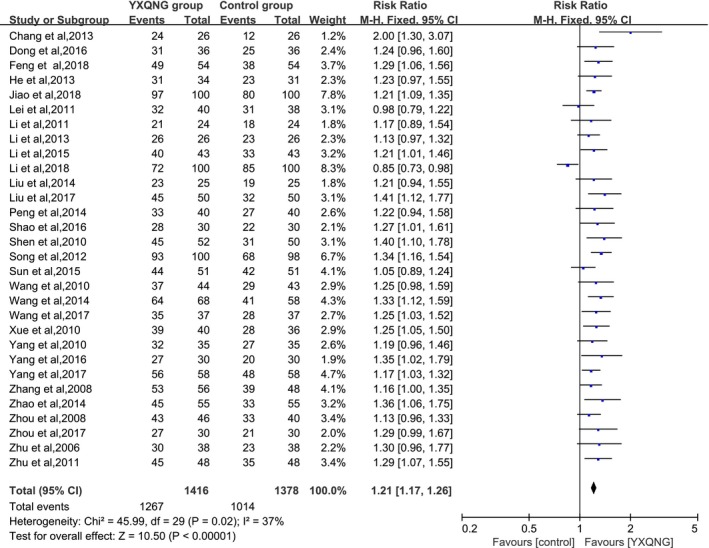
Effect of YXQNG on the improvement of symptoms in CCCI patients

In total, 30 trials evaluated the effectiveness of YXQNG in treating CCCI. The meta‐analysis indicated that the ratio between the combined RR of the total effective rate and the 95% CI was 1.21 (1.17, 1.26) (As shown in Figure 3), with no significant heterogeneity, suggesting that YXQNG could significantly improve the symptoms of CCCI.

#### 3.4.2 Secondary outcomes: the changes of blood viscosity and cerebral blood flow

**Figure 4:**
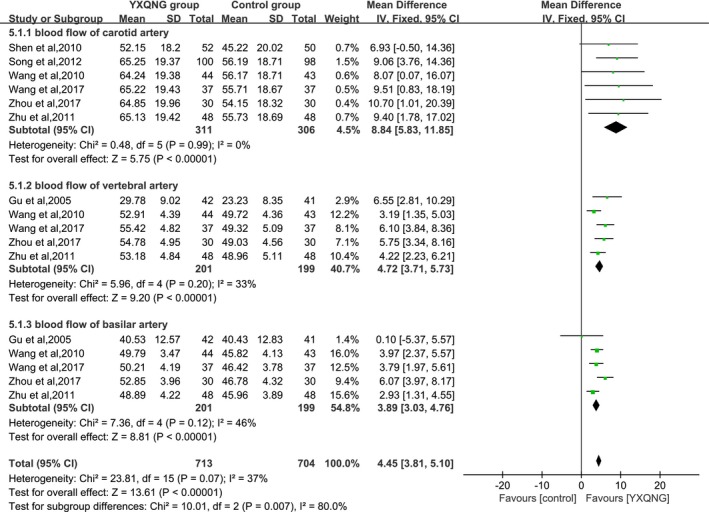
Effect of YXQNG on the improvement of cerebral blood flow rate in CCCI patients

**Figure 5:**
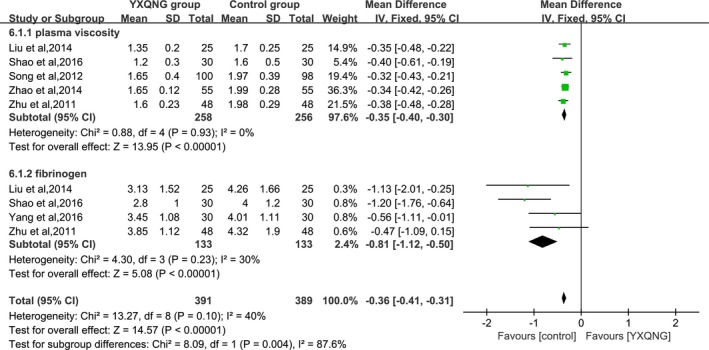
Effect of YXQNG on the improvement of blood viscosity in CCCI patients

The changes of cerebral blood flow rates due to the treatment of YXQNG were reported in 7 trials. The meta‐analysis indicated that the combined MD and 95% CI of blood flow of the carotid artery, vertebral artery, and basilar artery detected by TCD were, respectively, 8.84 (5.83, 11.85), 4.72 (3.71, 5.73), and 3.89 (3.03, 4.76) (As shown in Figure 4), meaning that YXQNG could significantly improve the blood flow rates of intracranial vascular. The changes in blood viscosity were reported in 5 trials. The combined MD and 95% CI of plasma viscosity and fibrinogen, respectively, were −0.35 (−0.40, −0.30) and −0.81 (−1.12, −0.50) (As shown in Figure 5).

### 3.5 Adverse effects

Among all the included studies, 15 studies described the adverse effects, of which only 2 studies reported that patients in the YXQNG group had the adverse effects of slight nausea and vomiting. The other 13 studies showed that there were no significant adverse reactions in the treatment group.

### 3.6 Publication bias

**Figure 6:**
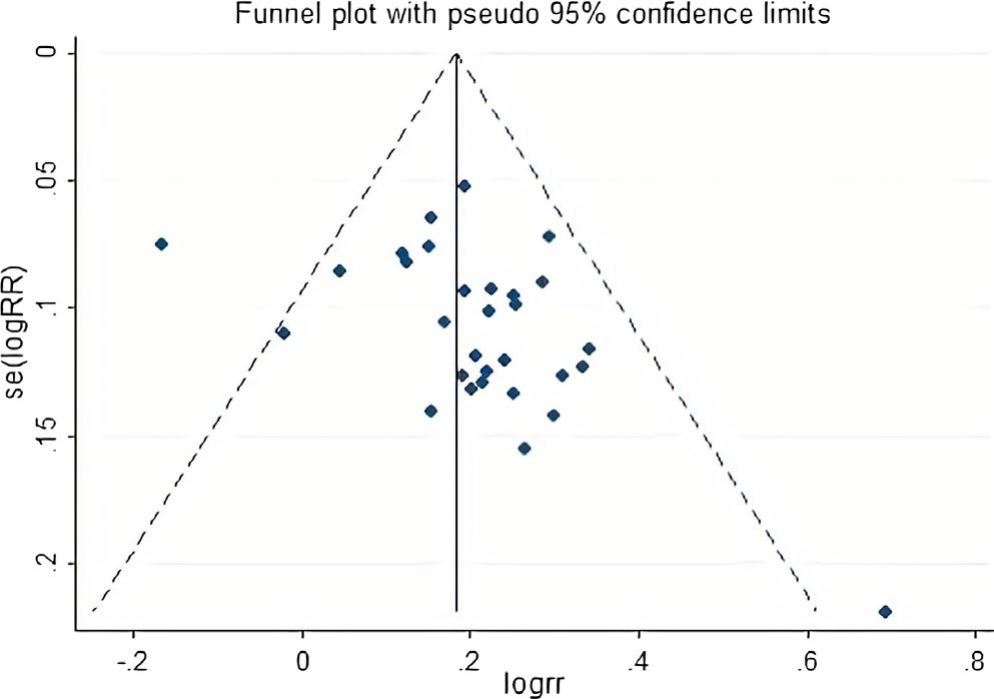
Funnel plot of the total effective rate of the treatment of CCCI patients with YXQNG

The funnel plots were asymmetrical on the visual inspection, suggesting the possible risk of publication bias (Figure 6). Moreover, the Begg's test revealed that *P*> |z| = 0.059, whereas the Egger's test result was *p* = .036, indicating that a certain extent of publication bias possibly existed.

## 4 DISCUSSION

### 4.1 The clinical efficacy analysis

The results of the meta‐analysis showed that the clinical effect of the experimental group was increased after using YXQNG, while the blood viscosity was reduced, and the blood supply to the brain was improved. Compared with the control group without YXQNG, the patients with CCCI could get a better clinical treatment effect by adding YXQNG on the basis of conventional treatment. The meta‐analysis results of the clinical total effective rate comparison have both clinical and statistical significance.

The latest Chinese epidemiological statistics of the Annual Epidemiological Report in 2016 showed that among elderly people over the age of 65, the prevalence of CCCI could be as high as two‐thirds, carrying enormous consequences for the economy and society (Calabrese et al., 2016). CCCI is an alarming condition leading to varied clinical manifestations of neurological deficits, particularly neuronal damage and cognitive impairment. However, the ideal treatment of CCCI is still a topic of debate, due to the lack of relevant high‐quality RCTs (Zhao, 2014; Zhou et al., 2018). This systematic review showed that YXQNG is potentially effective treatment for patients with CCCI, with improvements in symptoms including headaches, vascular cognitive impairment, dizziness, and sleep disorder. No serious adverse events were reported. In conclusion, YXQNG is an effective and safe management option for patients with CCCI, even for elderly ones. Our results are consistent with those of a previous, high‐quality randomized trial of YXQNG for CCCI, which demonstrated that YXQNG as a monotherapy could effectively improve the symptoms of CCCI (Chang, 2013).

The detailed pathological mechanisms of CCCI remain unknown. But, in general, CCCI is a result of cerebral vascular hypoperfusion, manifesting as a cerebral blood flow incapability, resulting in permanent cell death and nerve necrosis (Zhou, 2017). Normal CBF is about 50–55 ml/100 g brain tissue/min (Calabrese et al., 2016; Zhou & Liu, 2008). When CBF declines to the threshold value of 25–10 ml/100 g brain tissue/min, neuronal damage is obvious, leading to the early but reversible manifestations of cerebral infarction such as dizziness and headache (Zhou & Liu, 2008). Previous studies have also indicated that changes in blood viscosity are an important pathological mechanism of CCCI. Hyperviscosity, mainly induced by hyperfibrinogenemia and other reasons, can cause a significant decrease in cerebral blood flow (Liu, 2014; Peng, Chang, & Wang, 2014; Shao, Zeng, & Yang, 2016; Xue, Kan, & Ding, 2015; Zhang & Shi, 2008). The decline of cerebral blood flow leads to structural injuries in neuron and functional disorders in patients. In our review, the results revealed that YXQNG can act through both lower blood viscosity and improved cerebral blood supply to improve the symptoms of CCCI, consistent with the conclusions of previous studies (Shao et al., 2016; Zhou & Liu, 2008). In animal studies, evidence also suggests that YXQNG can lower the plasma and blood viscosity at the low or high shear rate, simultaneously improving the cerebral blood flow, which is in line with the clinical trials (Zhu & Liu, 2006; Zhu, Pan, & Tang, 2011).

### 4.2 Safety evaluation

Among the 15 studies included, only 2 studies reported mild nausea and vomiting in the patients in the YXQNG group. The other 13 studies showed no significant adverse reactions in the treatment group. It is suggested that YXQNG had good safety and fewer adverse reactions. At the same time, it is suggested that clinical research should pay more attention to drug safety in the future and improve the observation and report of safety indicators to increase the value of clinical research.

### 4.3 Methodology quality evaluation

Among all the studies included, none described allocation concealment, the blinding of participants and personnel, and the blinding of outcomes assessment. Also, only 14 trials reported both the curative effects and adverse effects objectively, while the other 16 studies only reported the effectiveness. The reliability of the evaluation conclusion was reduced by the influence of low‐quality factors. The existence of publication bias may also be a factor affecting the evaluation conclusion of the system. It is necessary to carry out a randomized controlled trial with reasonable design, standardized operation, multicenter designs, and large sample to improve the quality of the study to further verify the efficacy and safety of YXQNG in the treatment of CCCI. At the same time, it is suggested that the researchers should strictly carry out the experiment according to the standards to improve the quality of the experiment and draw more reliable conclusions.

### 4.4 Limitations

This systematic review had several limitations. First, the quality of the included studies was not particularly high. Second, all of the eligible participants were Chinese and it is unclear whether there will be the same research results in other countries and regions, which limits the application and promotion of YXQNG. Third, the follow‐up duration and the conventional treatment of the included studies were not particularly aligned, which resulted in heterogeneous findings. Lastly, as little information about the adverse effects could be obtained and, therefore, this review was unsuccessful in conducting a systematic review of the adverse effects.

## 5 CONCLUSION

This systematic review revealed that YXQNG could improve cerebral blood flow rates and the symptoms of patients with CCCI, with no serious adverse effects. Since the literature reviewed was affected by factors such as the lower quality of the included studies, the systematic evaluation's conclusion is not very reliable. Thus, more rigorously designed trials are needed.

## CONFLICT OF INTERESTS

The authors declare that they have no conflict of interests.

## AUTHORS’ CONTRIBUTIONS

Yujuan Jia and Jie Wang acquired data. Yujuan Jia and Yuli Hou drafted the manuscript. All authors take responsibility for the paper as a whole. All authors read and approve the final manuscript.

## ETHICS APPROVAL AND CONSENT TO PARTICIPATE

Not Applicable.

## Data Availability

We declared that materials described in the manuscript, including all relevant raw data, will be freely available to any scientist wishing to use them for noncommercial purposes, without breaching participant confidentiality.
